# Informed consent approaches for clinical trial participation of infants with minor parents in sub-Saharan Africa: A systematic review

**DOI:** 10.1371/journal.pone.0237088

**Published:** 2020-08-04

**Authors:** Angela De Pretto-Lazarova, Domnita Oana Brancati-Badarau, Christian Burri

**Affiliations:** 1 Department of Medicine, Swiss Tropical and Public Health Institute, Basel, Switzerland; 2 University of Basel, Basel, Switzerland; 3 Life and Health Sciences and Aston Brain Centre, Aston University, Birmingham, United Kingdom; University of Dschang Faculty of Medicine and Pharmaceutical Sciences, CAMEROON

## Abstract

**Background:**

Regulations are vague regarding the appropriate decision-maker and authority to consent for children of minor parents participating in clinical trials. In countries with high rates of underage mothers, such as in sub-Saharan Africa, this lack of guidance may affect the rights of potential paediatric participants already bearing increased vulnerability. It can also influence the recruitment and generalizability of the research. We provide evidence and discuss informed consent management in such cases to inform best practice.

**Materials and methods:**

We searched PubMed/MEDLINE, Embase, CINAHL, and Google Scholar for articles published up to March 2019. In total, 4382 articles were screened, of which 16 met our inclusion criteria. Studies addressing informed consent in clinical trials involving children with minor parents in sub-Saharan Africa were included. We performed descriptive and qualitative framework analyses. The review was registered in PROSPERO: CRD42018074220.

**Results:**

Various informed consent approaches were reported. Articles supporting individual consent by minor parents based on emancipation or “mature minor” status lacked evidence in the context of research. National laws on medical care guided consent instead. When no laws or guidance existed an interpretation of the local decision-making culture, including community engagement and collaboration with local ethics committees, defined the informed consent approach.

**Conclusions:**

The review emphasises that the implementation of informed consent for children with minor parents may be variable and hampered by absent or ambiguous clinical trial regulations, as well as divergent local realities. It may further be influenced by the research area and study-specific risks. Clear guidance is required to help address these challenges proactively in clinical trial planning. We provided a set of questions to be considered in the development of an ethically acceptable informed consent approach and proposed information that should be integrated into international clinical trial guidelines.

## Introduction

Enrolment of children into clinical trials (CTs) is mandatory to enable the development of new medicines for this population [1]. Infectious diseases and malnutrition remain essential factors affecting childhood mortality, with around 50% of all cases occurring in Africa [2–4]. Compared to Europe or the USA, a higher proportion of CTs conducted in sub-Saharan Africa (SSA) involve children [5, 6]. Adolescent birth rates in SSA countries are also among the highest worldwide [7], increasing the likelihood that research staff might have to deal with the ethical challenge of obtaining valid informed consent (IC) for infant participation from minor parents.

International guidelines on the conduct of CTs state that by being recognised as "emancipated" or "mature minors" through marriage, parenthood, etc. [1, 8], adolescents may be allowed to consent autonomously. However, it remains unclear whether autonomous consent refers only to adolescent’s own research participation or whether such minors may consent independently for their child as well.

Further specifications regarding the "emancipated" or "mature minors" status are subject to national provisions [1, 8, 9]. In the UK and the USA, professional guidelines recognise that minor parents can be responsible for medical decision-making for their children if they are considered competent. Nevertheless, these guidelines lack strict criteria or principles defining such competence in relation to minor parents and its applicability to clinical trials [10, 11].

In some SSA countries, such as Kenya, guidelines may be in place (e.g., a national CT regulation or institutional guidance for the conduct of CTs) determining whether minor parents may consent for their children [9, 12]. However, such guidance may be missing, unclear, or difficult to source in other SSA countries. Even when concepts for “emancipated” or “mature minors” exist in general national legislations or research specific guidelines, their transferability to the context of CTs and the consent for children of minors often remain unspecified [13]. In addition, social and cultural norms may differ from country to country posing challenges for researchers in the development and implementation of IC procedures [9, 14].

Considerable efforts in the past decades sought to improve quality standards in global paediatric research, including strengthening recommendations on IC practices [15–17]. However, formal international guidance on implementing an ethically acceptable approach to the IC process for children with minor parents in various CT contexts is still lacking. There is a paramount need for best practices on IC, which ensure adequate protection while maintaining the option to enrol children under such circumstances.

The objective of this study was to address this gap by mapping the reported approaches to IC in paediatric CTs involving minor parents in SSA as identified through a systematic literature review.

## Materials and methods

This review followed the PRISMA 2009 statement (S1 PRISMA Checklist) [18] and was registered in the PROSPERO database (CRD42018074220) [19].

### Search strategy

We conducted a systematic literature review and searched PubMed/MEDLINE, Embase, CINAHL, and Google Scholar to collect information on IC practices for children with minor parents included in CTs conducted in SSA. We used search terms related to the elements of IC, decision-making, CTs, minors, and SSA (Box 1). Reference lists of included articles were also screened. We performed the initial search in July 2017 (S1 Text) and updated it in March 2019 based on a reviewed search strategy by a medical librarian. The changes applied to the search strategy included: improving the combination structure of registered and free-text terms, removing language filters, removing animal studies, instead of limiting to humans, removing redundancies (term combinations were removed as single terms already covered them), complementing child MeSH and free text terms, as well as adding the terms “research”, “placebo”, and all sub-Saharan African countries. Information on search strategies for all other databases can be found in the appendix (S1 Text). No protocol was published for this review.

**Box 1. Search strategy (updated search)**

**Key elements**

Informed consent **AND** minors **AND** decision-making **AND** clinical trials **AND** sub-Saharan Africa

**PubMed**

("Informed Consent"[Mesh] OR "Parental Notification"[Mesh] OR "Presumed Consent"[Mesh] OR "patient information"[tiab] OR consent[tiab] OR consented[tiab] OR consenting[tiab] OR assent*[tiab] OR parental permission*[tiab])

**AND**

("Minors"[Mesh] OR "Child"[Mesh] OR "Infant"[Mesh] OR "Adolescent"[Mesh] OR "Child, Orphaned"[Mesh] OR "Pediatrics"[Mesh] OR "Pregnancy in Adolescence"[Mesh] OR "Maternal Age"[Mesh] OR "Vulnerable Populations"[Mesh] OR "Child Health Services"[mh] OR "Hospitals, Pediatric"[mh] OR "Intensive Care Units, Pediatric"[Mesh] OR minor*[tiab] OR pediatr*[tiab] OR paediatr*[tiab] OR child[tiab] OR children[tiab] OR childhood[tiab] OR infant*[tiab] OR newborn*[tiab] OR new born*[tiab] OR baby[tiab] OR babies[tiab] OR neonat*[tiab] OR perinat*[tiab] OR postnat*[tiab] OR kid[tiab] OR kids[tiab] OR boy*[tiab] OR girl*[tiab] OR preschool*[tiab] OR kindergar*[tiab] OR prepuberty*[tiab] OR prepubescen*[tiab] OR juvenil*[tiab] OR youth*[tiab] OR puber*[tiab] OR pubescen*[tiab] OR schoolchild*[tiab] OR highschool*[tiab] OR under-aged*[tiab] OR underage[tiab] OR teen*[tiab] OR adolescen*[tiab])

**AND**

("Parents"[Mesh] OR "Legal Guardians"[Mesh] OR "Caregivers"[Mesh] OR "Decision Making"[Mesh] OR "Judicial Role"[Mesh] OR "Mental Competency"[Mesh] OR "Comprehension"[Mesh] OR "Liability, Legal"[Mesh] OR "Personal Autonomy"[Mesh] OR "Child Welfare"[Mesh] OR "Infant Welfare"[Mesh] OR parent*[tiab] OR proxy[tiab] OR representative*[tiab] OR guardian*[tiab] OR caregiver*[tiab] OR care giver*[tiab] OR surrogate*[tiab] OR decision making*[tiab] OR capacity[tiab] OR capab*[tiab] OR competen*[tiab] OR legal-competen*[tiab] OR legally-competen*[tiab] OR matur*[tiab] OR emancipat*[tiab] OR waiv*[tiab] OR exempt*[tiab] OR autonomy[tiab])

**AND**

("Biomedical Research"[Mesh] OR "Clinical Trials as Topic"[Mesh] OR "Research Subjects"[Mesh] OR trial[tiab] OR trials[tiab] OR random*[tiab] OR RCT[tiab] OR placebo[tiab] OR research*[tiab])

**AND**

("Developing Countries"[Mesh] OR "Poverty"[Mesh] OR "Neglected Diseases"[Mesh] OR "Culture"[Mesh] OR "Culturally Appropriate Technology"[Mesh] OR "Global Health"[Mesh] OR "Health Resources"[Mesh] OR "Global Burden of Disease"[Mesh] OR low income*[tiab] OR low-resource*[tiab] OR resource*[tiab] OR resource-limited[tiab] OR resource-poor*[tiab] OR resource-restricted[tiab] OR developing countr*[tiab] OR global*[tiab] OR international*[tiab] OR developing world*[tiab] OR less-developed[tiab] OR less-advanced[tiab] OR poverty-related*[tiab] OR LMIC*[tiab] OR low-and-middle-income[tiab] OR angola[tiab] OR angolan[tiab] OR benin[tiab] OR botswana[tiab] OR "burkina faso"[tiab] OR "upper volta"[tiab] OR burundi[tiab] OR "côte d’ivoire"[tiab] OR "cote d’ivoire"[tiab] OR "ivory coast"[tiab] OR cameroon[tiab] OR camerun[tiab] OR kamerun[tiab] OR "central african republic"[tiab] OR chad[tiab] OR congo[tiab] OR zaire[tiab] OR djibouti[tiab] OR "equatorial guinea"[tiab] OR eritrea[tiab] OR ethiopia[tiab] OR gabon[tiab] OR gambia[tiab] OR guinea[tiab] OR "guinea bissau"[tiab] OR kenya[tiab] OR lesotho[tiab] OR liberia[tiab] OR malawi[tiab] OR mali[tiab] OR mauritania[tiab] OR mozambique[tiab] OR namibia[tiab] OR niger[tiab] OR nigeria[tiab] OR nigerian[tiab] OR rwanda[tiab] OR senegal[tiab] OR "sierra leone"[tiab] OR somalia[tiab] OR south africa[tiab] OR "south sudan"[tiab] OR sudan[tiab] OR swaziland[tiab] OR tanzania[tiab] OR togo[tiab] OR uganda[tiab] OR zambia[tiab] OR sambia[tiab] OR zimbabwe[tiab] OR rhodesia[tiab] OR "Africa South of the Sahara"[mesh]) NOT (animals[mh] NOT humans[mh])

We requested inaccessible articles from different libraries but did not contact authors. Articles and conference abstracts potentially relating to our topic, but for which a determination of eligibility was impossible without full-text access, were listed in the Supporting information (S1 Table). Books were rarely accessible and, therefore, completely excluded from the analysis. When available information on a book (accessible or inaccessible) suggested that it might relate to our search, the book was also listed in the Supporting information (S1 Table).

We did not limit our review to a particular study type and searched for any publication containing information about IC by minor parents in paediatric CTs conducted in SSA.

### Eligibility criteria and screening

We exported all search results to a reference management software (Endnote X7). After removing duplicates, we created an MS Excel table capturing selected information from the extracted literature (Author, Year, Journal/Publisher, Title, Abstract, Keywords, ISBN/ISSN, DOI, and URL). Two independent reviewers (ADP and DOB) received a copy of the excel sheet and first screened articles based on title and abstract according to predefined eligibility criteria (Box 2).

**Box 2. Eligibility criteria**

**Inclusion criteria**

Including any type of study, if relating to all of the following four key elements:

- Informed consent procedure (proxy decision-maker, autonomous consent, assent, preterm consent)
- Clinical trials (drug trials, vaccine trials, diagnostic trials, medical device trials, surgical trials, emergency research trials, nutritional supplementation trials)
- Children as
  - Participants (neonates, infants, toddlers, small children) with the age of 0.0–4.9 years
  - Minor parents (adolescents, teenagers, mature minors, emancipated minors) with the age of 12.0–17.9 years
- Sub-Saharan Africa (or global or international relevance, including sub-Saharan Africa)

**Exclusion criteria**

Excluding any type of study, if relating to:

- Adults
- Children with the age of 5.0–11.9 years
- Vulnerable participants in the broader sense (individuals with disabilities, geriatric subjects, ethnic minorities, migrants, etc.)
- Developed countries only
- Informed consent procedure in other than clinical trials:
  - Observational studies (with and without blood samples), quality of life studies, preventive interventions (health care/health behaviour/immunisation)
  - Reproductive health care (HIV testing, abortion, fertilisation, contraception, adoption, pregnancy, circumcision/sterilisation, etc.)
  - Biobanking, organ donation, blood transfusion
  - Genetic testing, new-born screening
  - Diverse treatments
  - Euthanasia/end-of-life decision-making
  - Surgery (as treatment)
  - Emergency treatment/treatment of serious illnesses
  - Nutritional studies, if only addressing natural behaviour, such as breastfeeding
  - If it is a study report using blood samples from a primary clinical trial
- Other informed consent topics, such as addressing exclusively:
  - Informed consent understanding
  - Informed consent return rates
  - Informed consent confidentiality issues
- Other language than English and French

When eligibility was unclear based on title and abstract, available full-texts were screened. The interrater reliability was moderate (Cohen’s Kappa *κ* = 0.47) for the initial screening and substantial (Cohen’s Kappa *κ* = 0.71) for the update search screening [20]. Disagreements between reviewers were mostly systematic and were all resolved in several rounds of discussion. One reviewer (ADP) performed the full-text assessment and a second reviewer (DOB) verified a random sample of 10%. Included papers’ full-text was screened systematically looking for minor parents using pre-defined search terms (Box 3).

**Box 3. Screening strategy**

**Full-text screening 1**

In case of missing key information after the title and abstract screening:

- Screen/Read pre-defined text sections (abstract, consent section, method section, and conclusion) and check if the *key elements* are addressed.
- Search for information about the *key elements* using the following pre-defined search terms:
  - “consent” OR “assent” OR “permi*”
  - “trial” OR “research”
  - “child*” OR “ped*” OR “paed*” OR “minor” OR “infant” OR “adolescent” OR “teen” OR “matur*” OR “parent” OR “mother” OR “father” OR “guardian” OR “repr*”
  - “inter*” OR “global” OR “countr” OR “develop*” OR “income” OR “resource”

**Full-text screening 2**

For all included papers after the title, abstract and full-text screening 1:

- Screen/Read pre-defined text sections (abstract, consent section, method section, and conclusions) and check if the topic of *minor parents of paediatric clinical trial participants* is addressed.
- Search for information about *minor parents of paediatric clinical trial participants* using the following pre-defined search terms:
  - “prox*” OR “surr*”
  - “consent” OR “assent”
  - “child” OR “adol*” OR “minor” OR “teen” OR “age”
  - “major” OR “eman*” OR “marr*”
  - “parent” OR “mother” OR “father”
  - “capa*” OR “compe*”

### Data extraction and analysis

For the included articles, we extracted characteristic information on study type and procedures, country, health conditions, and the medical interventions addressed. We performed a descriptive and qualitative framework analysis using MAXQDA (VERBI GmbH) and MS Excel [21].

### Critical appraisal of studies

Due to the information and study types identified in this review, no conventional assessment of bias risk or quality appraisal was applicable. We addressed the quality of the information descriptively in the results by reviewing the source and comprehensiveness of the implemented and recommended IC approaches, and did not exclude articles based on type or quality.

## Results

**Fig 1:**
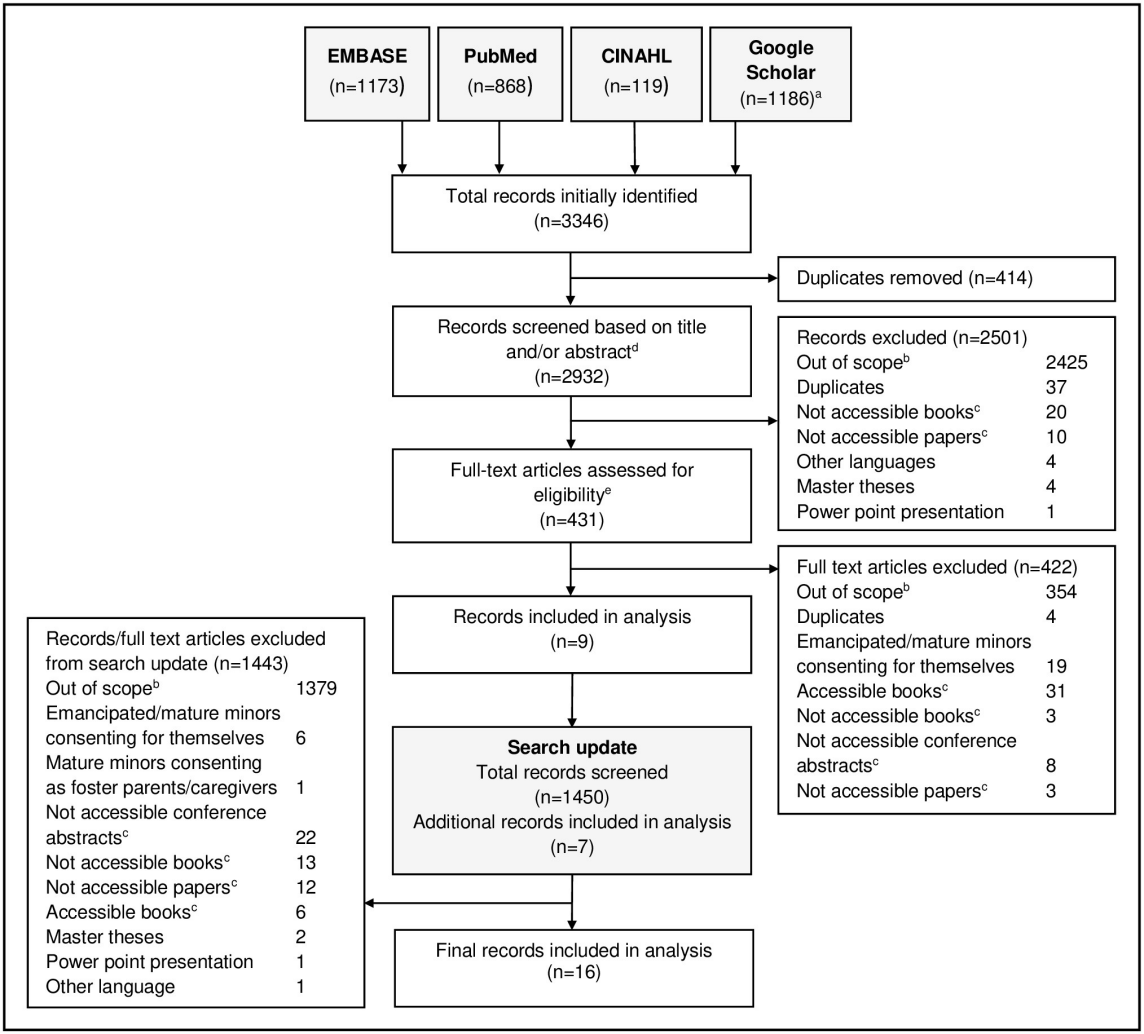
Study-selection flow diagram. ^a^The total number comes from three combined Google Scholar searches. ^b^Out of scope: not addressing informed consent, not addressing SSA, not addressing children < 5, not addressing clinical trials, not addressing minor parents, not clear if addressing minor parents. ^c^A list of these books/papers/conference abstracts can be found in the Supporting information (S1 Table). ^d^If information about the key elements of the search lacked in the abstract or title, articles’ full text was also screened using keywords (Box 3). ^e^Full-text assessment was done based on a secondary screening using keywords relating specifically to minor parents (Box 3).

We initially identified 3346 articles from the literature search (Fig 1). After removing duplicates (n = 414), we screened the titles and abstracts of 2932 articles, and when eligibility remained unclear, we screened the full-text. 2501 articles were excluded, and the full-text of 431 was assessed, resulting in 9 articles included in the analysis. Our search update found 1450 additional articles from which seven were eligible, amounting to a total of 16 articles. The reasons for exclusion were: out of scope, emancipated/mature minors consenting for themselves, not their children, duplicates, languages other than English and French, master theses, PowerPoint presentations, books, and non-accessible full-texts of conference abstracts and papers.

The 16 identified articles included various study types (Table 1) categorised into: case studies (n = 4) [22–25], one of which included a review [24], reviews of national legislations and ethical discussions (n = 4) [26–29], reviews of IC challenges (n = 3) [30–32], meeting/workshop reports (n = 3) [33–35], and mixed-methods research (n = 2) [36, 37]. Nine studies addressed six particular SSA countries (Senegal, Côte d’Ivoire, Kenya, Botswana, South Africa, and Uganda), while the others related to SSA, low- and middle-income countries, or global CTs in general. Infectious diseases were the most prevalent health conditions, and vaccines were the most frequently discussed medical intervention. Five articles were secondary publications of CT experiences [22–25, 36] and described the IC approach for children with minor parents applied in specific CTs [38–42]. Five further articles discussed the national legislation concerning IC requirements, including in the case of minor parents [26–29, 37]. The remaining articles mentioned IC by minor parents among several ethical challenges faced in clinical research conducted in developing countries [30–35].

**Table 1:** Study characteristics.

#	Study	Study type	Country	Health condition	Medical intervention	Content
1	Diallo et al. (2003) [22]	Case study	Senegal	Alternative nutrition/Malnutrition	Nutritional supplement	Practical informed consent experience in a specific CT [38]
2	N’Goran et al. (2019) [23]	Case study	Ivory Coast	Schistosomiasis	Drug	Practical informed consent experience in a specific CT [39]
3	Ott et al. (2019) [24]	Case study and review of national legislation, Ethical discussion and consensus by an international panel	Global (CT example in Latin America, but including African view)	Unspecified (CT example of Clinical Otitis Media and Pneumonia)	Unspecified (CT example about a vaccine)	Practical informed consent experience in a specific CT [40] and IC recommendations
4	Preziosi et al. (1997) [25]	Case study	Senegal	Pertussis	Vaccine	Practical informed consent experience in a specific CT [41]
5	Slack and Strode (2016) [26]	Review of national legislation, Ethical discussion	South Africa	Unspecified (example of HPV vaccine trial with adolescents)	Unspecified (example of HPV vaccine trial with adolescents)	Proxy consent recommendations for CTs
6	Strode et al. (2014) [27]	Review of national legislation, Ethical discussion	South Africa	HIV	Drugs and vaccines	Recruitment challenges with adolescents in CTs
7	Strode and Slack (2011) [28]	Review of national legislation, Ethical discussion	South Africa	Unspecified (Research with more than a minor increase over minimal risk)	Unspecified (Research with more than a minor increase over minimal risk)	Parental informed consent responsibilities in CTs
8	van Wyk (2003) [29]	Review of national legislation, Ethical discussion	South Africa	HIV	Vaccines	Informed consent rights for minors in CTs
9	Colom and Rohloff (2018) [30]	Scoping review	Low- and middle-income countries	Unspecified	Unspecified	Cultural informed consent challenges in CTs
10	Idoko et al. (2016) [31]	Review and experience report	Sub Saharan Africa	Infectious diseases	Vaccines	Informed consent challenges in CTs
11	Lema et al. (2009) [32]	Review and experience report	Sub Saharan Africa	HIV/AIDS, cancer, diabetes, hypertension and congenital anomalies/congenital disabilities	Drugs and vaccines	Informed consent challenges in CTs
12	Mamotte et al. (2010) [33]	Meeting report	Sub-Saharan Africa	HIV/AIDS, tuberculosis, and malaria	Vaccines	Ethical challenges in CTs
13	Ravinetto et al. (2010) [34]	Workshop and meeting report	Sub-Saharan Africa (example of Uganda)	Tropical diseases	Unspecified	Ethical challenges in CTs
14	van Hoog (2013) [35]	Report	Global	Infectious diseases	Vaccines	Recruitment challenges with young people in CTs
15	Angwenyi et al. (2014) [36]	Mixed methods (IDI, FGD, survey, observations, document reviews)	Kenya	Malaria	Vaccine	Practical informed consent experience in a specific CT [42]
16	Kasule (2013) [37]	Mixed methods study (PhD thesis: cross-sectional exploratory study)	Botswana	HIV	Drugs and vaccines	Practical informed consent experiences in CTs

CT, Clinical trial; FGD, Focus Group Discussion; IDI, In-Depth Interview.

One article [30] was a review, which included another of the included articles [24]. We considered it for analysis only when it provided additional information.

We systematically extracted information on minor parents according to six themes: The frequency and age of these parents, the IC approach and the related references, IC challenges, and recommendations affecting the IC approach (Table 2).

**Table 2:** Framework of themes concerning minor parents.

#	Study	Frequency of minor parents	Age of minor parents (years)	IC approach with minor parents	References linked to the IC approach with minor parents	Challenges addressed concerning IC with minor parents	Recommendations with potential influence on the IC approach with minor parents
1	Diallo et al. (2003) [22]	2 (1.4%) of CT participants had a minor mother	15 and 16	IC provided by minor mothers. However, the father or paternal grandparents were included in the information process and contributed to decision-making. Only with the mother’s authorisation was the child included. The primary CT report states “oral informed consent was obtained from all mothers of the study infants” [38].	No source referenced.	The child’s father and grandparents did not consider the minor mothers as mature enough to decide on the research participation of children. The practicability of this IC approach is, therefore, questionable. The study was conducted without an ethical approval: A local EC/IRB did not exist, and an EC in the Sponsor’s country was not consulted as no EC had experience with research in developing countries; the local Ministry of Health approved the protocol.	No recommendation provided.
2	N’Goran et al. (2019) [23]	Unspecified	< 21	IC not provided by minor mothers. Although married minors are considered emancipated under local law, they could not provide consent for their children, as decided by the national ethics committee. However, it remains unclear to what extent the minor parent participated in decision-making (e.g., assent). No CT report published yet.	The reference provided for the Ivory Coast minority law (1970) [43] addresses emancipation of minors through marriage, however, it does not explicitly refer to emancipated minor’s right to consent for their child’s participation in CTs.	No clear interpretation of the rights afforded by the “emancipated” status for minors versus the legal age of majority was provided in the local law. The EC decided that emancipated minors could not consent.	Involving fieldworkers with knowledge of the local population could facilitate the study recruitment. Revision of the ICH-GCP guidelines to reflect the flexibility and allow for adaptation to local settings.
3	Ott et al. (2019) [24]	211 (3%) of CT participants had a minor parent	< 18	IC was first provided by minor parents alone. During the CT, the IC procedure changed, and an additional consent was required by either the other parent (if an adult), or by the grandparents. Re-consent by the minor parent alone was sought once the age of majority was reached. The primary CT report (conducted in Latin America) addresses minor parents [40].	No source referenced.	No clear local laws or guidance on the IC process in the case of minor parents existed. The study was reviewed and approved by national public health authorities and a local EC, but after a routine review, the EC changed its requirements for the IC approach.	The EC took a decision based on the local cultural norm. The authors conducted a review of relevant literature and provided detailed recommendations: Involve local institutions, ethics committees, and community stakeholders proactively in the definition of the IC process, respect the minor parents and involve them in decision-making, include another decision-making party depending on cultural context and the minor’s capacity, implement a careful, adapted consent process, mitigate additional vulnerabilities of children of minor parents, and community engagement for a better understanding of the local context.
4	Preziosi et al. (1997) [25]	85 (4.1%) of CT participants had a minor mother	< 18	IC provided by minor mothers. The IC is explicitly considered valid. The primary CT report states “…and those whose parents agreed were vaccinated” [41].	No source referenced.	No challenges mentioned. As local EC/IRB did not exist, the protocol was reviewed and approved only abroad by an EC affiliated with the collaborative study and the Human Subjects IRB of the CDC.	Clinical trials in developing countries should be reviewed by a sponsor’s EC (if external) and a local one. Subsequent studies had, therefore, involved locally established “ad hoc” committees.
5	Slack and Strode (2016) [26]	Unspecified	< 18	IC should not be provided by minor mothers. When the mother is under the age of 18, her mother (the child’s grandmother) will be the child’s guardian. There is no information on whether the marital status of minor mothers makes a difference to this approach and how to handle IC when minor mothers have lost the support of their parents (as mentioned in earlier articles by the same authors, *see Strode & Slack 2011*, *2014*).	The sources refer to South African CT regulations (2013) [47] and a position paper about guidelines for the involvement of adolescents in research [51]. Neither source confirms the IC approach explicitly. The South African CT regulations generally state that “any IC given to the research must be in line with public policy” and non-therapeutic research with minors needs additional ministerial consent. The position paper for research with adolescents states that mature or emancipated minors (married or in military service) can consent for themselves. It also states that “other minors authorised to consent may include those who are parents”. But it does not explicitly address IC for the CT participation of their children.	There are no specific challenges mentioned.	The authors provide recommendations aimed at researchers who want to implement parental/guardian consent, which is distinguished from caregiver consent. Its implication on IC by minor parents is not addressed.
6	Strode et al. (2014) [27]	Unspecified	< 18	The legal competency of a married or unmarried, underage mother to consent is not evident (Strode and Slack 2011 provide more information). It is vaguely suggested that IC can be provided by minor mothers if they are married. The consent right in the case of unmarried minor mothers who have lost the support of their parents appears uncertain. Apparently, unmarried minor mothers used to consent as caregivers (especially when they had lost the support of their parents). As such caregiver consent is not accepted anymore according to a reform of the National Health Act; children of such mothers might be excluded from future research.	The source referenced [50] does not explicitly address minor parents consenting to CT participation of their children. The IC approach seems to be based on a conclusion by the authors that the restriction of the law around adolescent participation in research will affect children of minor mothers who lost the support of their parents and subsequently lose their right to consent as caregivers.	A law reform (Section 71 of the National Health Act) limits proxy consent to parents or legal guardians, excluding IC by caregivers. The authors highlight that “this principle will also apply to mothers under the age of 18 who have lost parental support …”	The authors criticise the new restrictive legislation to be reducing adolescents’ access to research participation while promoting an IC approach that is adapted to the research setting.
7	Strode and Slack (2011) [28]	Unspecified	< 18	IC provided by minor mothers if they are married. IC provided by maternal grandmothers if the minor mothers are unmarried and the father of the child has no parental responsibilities and rights.	The South African Children’s Act (2005) [46] is clear about the minor mothers’ guardian being also the guardian of the minor mother’s child when she is unmarried (and in case the father has not guardianship rights). According to a legal review of the South African law [53], minor mothers are recognised as their child’s guardians once they are married. This source was inaccessible, and the statement could not be confirmed. Strode and Slack also state that as guardians, married minor mothers can then consent to “all forms of research” on behalf of their child. This is the authors’ interpretation, as no source is referenced. The South African Children’s Act (2005), does not explicitly state that this IC regulation also relates to research participation.	Local laws (Children’s Act and the National Health Act) and ethical guidance are inconsistent concerning children’s capacity to consent to research as well as adults’ authority to provide proxy consent.	Based on a review of South African legislation, the authors conclude that “a biological mother, as the child’s legal guardian, has the authority to consent to all forms of health research on behalf of the child as long as she is 18 years or older, or under the age of 18 and married."
8	van Wyk (2003) [29]	Unspecified	< 21 (at time of study, currently: < 18)	Minor parents above 14 may provide consent for therapeutic research. Minors over 18 may also consent for non-therapeutic research (preventive HIV vaccine trials are considered non-therapeutic research). Proxy consent in non-therapeutic research is, however, limited by requiring consent by a legally authorised representative, assent by the participant and no more than negligible research risk (preventive HIV vaccine trials are considered more than negligible risk).	The South African Child Care Act [45] confirms that: 1) Minors over 14 may consent for themselves and their children for “any medical treatment”. The authors interpret medical treatment as interventions that benefit the individual patients and, therefore, extend the definition to “medical research of therapeutic nature”. 2) Minors over 18 may consent for “any type of operation.” The Authors extend this to cover also non-therapeutic research. However, consent for their children is not explicitly mentioned. (The referenced law is outdated and was replaced by the South African Children’s Act No 38 of 2005 in 2007 [46])	Under South African legislation, there is no consistent approach regarding the minor persons’ capacity to consent to research. There are inconsistent definitions of minors in law: e.g., the age of majority is reached at 21, but children are defined as minors under the age of 18. Restrictions for proxy consent for non-therapeutic research bearing a more than negligible risk result in a prohibition to include infants in such research.	The authors advise that research enrolment in non-therapeutic trials bearing more than negligible risk should be restricted to participants above the age of 21.
9	Colom and Rohloff (2018) [30]	Unspecified	Unspecified	The study addressing minor parents in this review refers to an included paper (*see Ott et al*. *2018*).	*See Ott et al*. *2018*	*See Ott et al*. *2018*	Local ethics committees are expected to play a substantial role in defining adaptive processes for IC. The community should be involved, and the local sociocultural context should be considered.
10	Idoko et al. (2016) [31]	Unspecified	< 18	IC provided by minor mothers. This approach is commonly allowed in most settings in Africa when the minor mothers are considered as mature or emancipated minors. However, some sponsors don’t agree with this definition.	There is no source referenced for the specific settings in Africa defining IC by mature or emancipated minors.	The authors state that there is “considerable debate” about the possibility of minor mothers to consent for themselves and their children in vaccine trials. It seems the issue is that some sponsors resist going along with local customs around this aspect. The issues perceived by sponsors concerning *minor mothers being allowed to consent for research participation of their children in most settings in Africa when considered as mature or emancipated minors*, are due to "legal reasons and may thus result in discrimination of this segment of the population."	Sponsors should give more consideration and leeway to the local culture while ensuring that the children’s rights are safeguarded. Regulatory authorities should be informed of the IC practice agreed upon by the sponsors and local researchers.
11	Lema et al. (2009) [32]	Unspecified	Unspecified	Not defining if IC by minor parents is acceptable for research. Only acknowledging the possibility of consent by minor mothers. However, researchers faced the scenario when consent from mothers (not specified if adult) with legal guardianship was later withdrawn by fathers, challenging the mother’s authority and the research team’s decision not to consult fathers. Also, elders in the family may generally”have to be consulted to give their nod before one can consent.”	No source referenced.	Cultural norms (i.e., patri- or matrilineal hierarchies) or a lack of legal clarification (e.g., individual parental rights, marital status, variations in research and medical care) may complicate the IC requirements for women in general and impede them from being able to consent autonomously. The authors highlight the paradox that minor mothers may not be considered able to consent autonomously for their own research participation, while at the same time being considered the legal guardians of their children enabled to consent to medical care.	Researchers should respect local social and cultural norms and values to prevent conflicts with the local communities. The IC process should be evaluated individually for every CT to potentially detect specific issues. This process should be budgeted for.
12	Mamotte et al. (2010) [33]	Unspecified	Unspecified	Not defining if IC by minor parents is acceptable. Only acknowledging that the case of minor parents is possible when obtaining proxy consent.	No source referenced.	Proxy consent in the case of minor parents may pose challenges.	Proxy consent in the case of minor parents requires special consideration.
13	Ravinetto et al. (2010) [34]	Unspecified	Unspecified	IC provided by minor mothers is accepted. The authors base this approach on the Guidelines of the Uganda National Council for Sciences and Technology, evaluating “adulthood” beyond age-dependence.	The Ugandan research guideline [48] does not confirm this approach. It states that emancipated minors (including minor parents) can consent for themselves, but it does not explicitly address IC for the CT participation of their children.	The authors consider the issue of minor parents as "more specific challenges" in relation to IC validity.	The decision whether minor parents are allowed to consent needs to balance social, cultural and legal factors. The definition of “adulthood” should be based on more than just a person’s age. The authors recommend involving social scientists and anthropologist in the development of effective, relevant, and ethical research and IC tools.
14	van Hoog (2013) [35]	Unspecified	< 18 (mostly, but global range 14–21)	IC provided by minor mothers for their children is recorded as accepted by most countries as these mothers are considered legally mature minors.	The source referenced [52] for this approach is not clear. It states that minor parents can be considered mature minors and consent for their own CT participation. It is not explicitly addressing CT participation of their children. Also, the approach is based on a personal communication concerning research in the USA.	The authors highlight that parental consent could be a barrier to research participation of young people.	Legal barriers need to be overcome, and youth organisations should be involved. Research advisory boards should also reflect and represent the specific target population. The IC process should be adapted to the needs of the participants or parents.
15	Angwenyi et al. (2014) [36]	Unspecified	16–17	No explicit statement on who gave informed consent in the case of minor parents. The primary CT report states "Written informed consent was obtained from the children’s parents or guardians" [42].	No source referenced.	No challenges mentioned.	No recommendation provided.
16	Kasule (2013) [37]	Unspecified	< 21 (at time of study, currently: < 18)	IC might theoretically be provided by minor parents if considered as mature minors. However, due to a lack of clear guidance, the IC was not provided by minor parents. A parent below the age of majority needed to be accompanied by an adult. It remains unclear to what extent the minor parent was enrolled in decision-making (e.g., assent).	The reference provided for mature minors did not address minor parents [49]. The Botswana Children’s Act (2009) [44] does not explicitly relate to emancipated/mature minors or IC for CT participation of their children.	Botswana does not have a national law on research with children, or a law on the concept of ‘mature’ or ‘emancipated minors’. Also, various legal acts in Botswana contain different concepts of ‘child’, ‘parent’, and ‘guardianship’ and different laws state different ages for childhood. (the Children’s Act now states clearly that whenever there is a conflict between the Children’s Act and other legislation/regulation, then the Children’s Act has to be considered unless it would harm the child). Due to a lack of clear guidance, IC was sought from representatives of the minor parents.	Clear research guidance for the legal and cultural context of Botswana is needed.

CT, Clinical trial; IC, Informed consent; IRB, Institutional Review Board; EC, Ethics Committee; CDC, Centers for Disease Control and Prevention; ICH, International Council of Harmonization for Technical Requirements for Pharmaceuticals for Human Use; GCP, Good Clinical Practice.

Three of the articles relating to specific CTs mentioned explicitly the number of children with minor parents ranging from 1.4 to 4.1% [22, 24, 25]. The legal age of majority was 18 in most countries, except for Côte d’Ivoire where it was 21 (Botswana and South Africa had recently changed from 21 to 18) [47, 54]. One article highlighted that the legal age of majority might range from 14 to 21 globally [35].

In five articles, minor parents were allowed to consent for research participation of their children [22, 25, 31, 34, 35]. In four articles, consent by minor parents was denied [23, 24, 26, 37]. One of these articles reported that researchers first allowed minor parents to consent for the CT, but later reconsidered their procedure, as the ethics committee changed its policy during the course of the CT [24]. Three other articles proposed conditional approaches to consent by minor parents, e.g., depending on the research risk or the marital status of the mothers [27–29], and two more articles simply acknowledged that the case of minor parents is possible and may pose challenges [32, 33]. One article involved a CT including children of minor parents, however, it did not report any details on the IC approach [36].

A majority (n = 11) of articles addressed challenges in designing an appropriate IC approach for CTs involving children with minor parents [22–24, 28, 29, 31–35, 37]. Most of these articles highlighted a lack of or inconsistency in local laws and guidance on the rights of minors in relation to clinical research [23, 24, 28, 29, 31, 32, 37]. A further challenge addressed in two older articles was a lack of local ethical review, as no local ethics committee existed at that time [22, 25]. Hence, one study was only reviewed by a foreign ethics committee and IRB [25], while the other was additionally submitted to the local ministry of health [22]. In the latter study, researchers experienced challenges when asking minor mothers to provide formal consent, as families viewed these mothers as immature; this resulted in involving the grandparents or fathers in the decision-making [22]. Two further articles addressed another challenge posed by a South African law reform restricting consent for children to adult parents and legal guardians, excluding other caregivers [26, 27]. This reform resulted in a specific barrier to recruiting children whose parents were minors and had lost the support of their parents [27].

Several articles provided specific references to IC approaches proposed for children with minor parents, such as four national laws on children’s rights [43–46], one national research regulation [47], one national research guideline [48], and four position papers on children’s rights in research [49–52]. One source referred to a legal review inaccessible for our study [53]. Based on these references, none of the applied or proposed IC approaches reported in the initial articles could be confirmed as mandatory or an established standard of practice. Some references mentioned conditions for adolescents’ autonomous consent for their own research participation without explicitly invoking their children [43, 48, 51, 52]. The only reference directly referring to consent approaches for children of minor parents was the South African Children’s Act (and its predecessor, the South African Child Care Act) [45, 46]. However, none of the national laws on children’s rights (including the South African ones) addressed clinical research [43–46].

Overall, only two articles provided comprehensive recommendations on IC for CT participation of children with minor parents [24, 28], as presented in detail in Table 2. One of these articles based the IC approach on a review and discussion of national legislation of children’s rights [28]. The other one addressed ethical considerations for the development of an IC approach for children of minor parents based on a review of relevant literature and guidelines, including consensus by an international expert panel [24].

Other articles offered general recommendations concerning IC implementation in SSA [23, 25–27, 30–32, 34, 35, 37]. These involved a consideration for the local context and norms [23, 27, 31, 32, 34], the capacity of local ethics committees and regulatory authorities [30, 31], the availability of context-adapted research guidelines and laws [26, 37], and the representation of target populations in research advisory boards [35].

Finally, we provide an overview of additional challenges for IC in research in SSA (Table 3) mentioned across the 16 articles. These are general considerations, which may benefit the development of an appropriate IC approach for CTs involving children with minor parents in resource-limited settings (RLS).

**Table 3:** Additional considerations for informed consent in sub-Saharan African research.

Theme	Issue
**Additional vulnerabilities of children with minor parents**	Increased poverty (including less access to health care, less educated parents) Interpersonal complications through possible child marriage Greater power differential between the minor parent and the researcher Less decision-making experience Impact of stressful conditions (child suffering from a chronic disease) on decision-making capacity
**Research risk**	Consent requirements may vary according to the research risk Research with minimal risk/a minor increase over minimal risk may allow for consent from caregivers Increased risk research requires consent by one or both parents or legal guardians
**Legal aspects**	Many developing countries experience a cultural transition Variable laws across and within countries (e.g., Common Law and Customary Law) The population may lack legal records, such as birth certificates and identification documents
**IRB/EC Approval**	ECs need to know relevant international and national regulations The review by the ECs varies from country to country Local laws and guidelines have to be considered When there is no law or guidance, relying on the decision by the ECs according to their ethical norms Studies sponsored from abroad should undergo dual review by local ECs and ECs abroad
**Community stakeholder**	Community engagement and pre-trial design efforts may be needed to set-up research activities Initial consent from local community stakeholders, leaders, or other key decision-makers may be needed
**Decision-making culture**	Communal, family and/or individual consent may be the norm Consultation with spouses or close family members may be needed Variable norms across and within countries (e.g., rural and urban areas)
**Gender dynamics**	Mothers and fathers may have different decision-making authority Determinations of decision-making authority may depend on the social structure (i.e., patri- or matrilineal) Mothers may have to consult their husbands, parents, or other family members (e.g., maternal uncles) Silent refusals by delaying research procedures are possible Biological fathers may not automatically also be the legal guardians of their children The marital status for both men and women may be criteria for the assignment of parental authority
**Assent**	The ability to assent depends on a child’s maturity and is recognised at different ages in various countries This concept of shared decision-making may not be consistent with local norms
**Autonomous consent by adolescents**	The balance of adolescents’ privacy needs and the demand for parental consent poses difficulties Parental consent may represent an obstacle to adolescent research participation (e.g., in sexual health research due to stigmatisation) Minors may be allowed to consent for themselves when they are considered mature or emancipated
**Caregivers vs legal guardians**	Unclear if consent by a caregiver is acceptable in some countries and under what circumstances The effort required to distinguish between parents, legal guardians, and caregivers is unclear Due to a lack of legal records, special precautions may be required, such as village chiefs confirming the identity of people
**Orphans**	Orphans are increasingly recognised as a special research population in developing countries in terms of HIV risk and transmission There is an ambiguity in defining the right decision-maker in the case of orphans and their children (e.g., consent by a High Court) Ambiguity may lead to the exclusion of orphans and their children from research for convenience reasons

IRB, Institutional Review Board; EC, Ethics Committee.

## Discussion

This systematic literature review presents evidence on CT recruitment and IC practice for children with minor parents in SSA. Overall, our results show that researchers experienced the need to find a solution concerning IC when enrolling children with minor parents and were challenged by the lack of a specific regulation or guidance.

A similar number of articles accepted or denied minor parents providing independent IC for their children and both approaches involved specific uncertainties. Becoming an emancipated or “mature minor” was the key argument promoting independent consent by minor parents [31, 34, 35]. When considering the referenced literature, however, we found this approach to lack legislative clarity and generalisability [43, 48, 51, 52]. First, the emancipation or “mature minor” status did not explicitly relate to clinical trials with children of minors. Instead, it related either to autonomous consent by adolescents for their own research participation or to medical care rather than research. Further, the conditions to reach emancipated or “mature minor” status, through marriage, parenthood, etc., vary across countries, as do the rights ensuing from the respective status. In N’Goran et al., minor parents could be considered emancipated when married; however, they could not provide consent for their children’s research participation [23]. Lema et al. mention that minor parents may have the authority to consent for their children’s medical care while not being considered mature enough to consent to their own research participation autonomously [32]. A recent position paper by the American Academy of Pediatrics confirms this ambiguity: All (US) states accept medical decision-making by minor parents for their children, without necessarily acknowledging minor parents as emancipated or mature to authorise their own medical care [11]. Another perspective is yet added by the Guidelines for Conduct of Clinical Trials in Kenya, which consider minor parents directly as “emancipated minors” able to consent for themselves and being explicitly allowed to consent to CT participation of their children [12]. These examples indicate that the legal status alone does not always equate to an adolescent’s capacity for decision-making and emphasise the need for clear conditions establishing minor parents’ competence to consent for themselves and their children.

In studies where minor parents were not considered emancipated or competent to consent independently for their children, consent was provided by an adult proxy [23, 24, 37], which included the other parent (if an adult), grandparents, or legally authorised representatives or guardians. This approach raises the problem of identifying appropriate decision-makers, a known issue for paediatric research in the SSA context [30]. It involves the additional consideration of gender dynamics, hierarchical family structures (e.g., matrilineal, or patrilineal), or shared versus individual decision-making within the family or community [30, 32, 34, 35, 37]. Also, formal identification of individuals accompanying children may pose problems, as people in RLS may lack birth certificates or identity documents [23, 26]. In one study, the village chief was therefore asked to confirm identities [23]. Ignoring local norms may affect the IC validity and, hence, the protection of CT participants and could result in a recruitment failure or subsequent consent withdrawal [32]. Therefore, community involvement in the development and approval of the IC approach before CT implementation is essential to address specific scenarios upfront and find practical and acceptable solutions.

CT participation risks may further influence the IC approach. Risks play a role in deciding whether one or both parents have to provide consent and at what age a person is capable of consenting. Earlier interpretations of South African laws restricted non-therapeutic trials bearing more than a negligible risk to participants above the age of 21 [29]. The research area may also have an influence, and in certain fields, such as HIV transmission prevention, additional consent by grandparents may pose a barrier to research participation of minor parents and their children, due to privacy reasons and fear of stigmatisation [35]. Hence, individual consent by minor parents alone might be encouraged to improve access to such research. Independent consent by minor mothers might also be encouraged in cases when children are typically accompanied by their mothers and health facilities are difficult to access [9, 22]. Requiring these mothers, when competent, to always consult their husbands or families before being able to consent, may be disruptive to the recruitment of these children. At the time when communities are informed about the CT, however, willingness to participate in the CT can also be discussed in advance, particularly in families where such a situation is expected.

In the case of consent by adult proxies, included studies lacked information on the extent of minor parents’ involvement in the IC process. Only one article mentioned explicitly how minor parents were consulted in parallel with the consent of an adult. It proposed that minor parents could first provide a co-consent and then re-consent independently when reaching majority during the CT [24]. This approach is supported by acknowledgements across literature in the past decade that minors should be involved in decision-making according to their developmental capacity [55, 56].

We further detected limited transparency for reported IC procedures for children of minor parents in primary CT publications. This is emphasised by the fact that we did not identify any primary CT publication addressing minor parents in our results. Five included articles, which were secondary studies on CT experiences, however, referenced primary CT publications. We reviewed these publications, and in three of them, we could not find any indication of the parents’ ages, and the IC statement was limited as well [38, 41, 42]. Minor parents’ involvement was only evident in the secondary studies’ publications [22, 25, 36]. One of the three primary CT publications stated that “oral IC was obtained from all mothers of the study infants”, and more information on minor mothers’, parents’ and husbands’ participation in decision-making was reported as significant only in the secondary study [38]. The second primary CT publication stated “those whose parents agreed were vaccinated” without mentioning that some of the consenting parents were minors [41]. The third primary CT publication provided the following statement: “written IC was obtained from the children’s parents or guardians” [42]. It is debatable how much more information beyond such blanket statements should researchers report in primary CT publications to effectively describe the IC procedures applied, considering typical word limitations in publishing and the relevance of the topic in relation to other information provided in CT publications.

### Strengths and limitations

This review has some limitations. Information about minor parents was scarce and typically included as a tangential thought only. Hence, we additionally developed a full-text screening strategy to increase our screening efficiency (Box 3). This strategy may have led to overlooking some relevant terms and articles limited to these terms. We identified many articles, representing secondary studies based on primary CT publications, which sometimes included minor mothers. Most of these primary CT reports, however, did not figure in our search results independently, probably due to lacking specific links to standardised keywords. As many of those primary CT publications also lacked a reference, we systematically excluded them, except when including the secondary studies, then we also considered the information provided in the primary CT publications, if accessible.

Further, we used Google Scholar to access also grey literature, such as dissertations, organisation reports, government publications, etc. The translated search, however, yielded more articles than Google Scholar was able to display, as it is limited to a maximum of 1000 articles [57]. We decided to include all accessible articles and ran two additional, very limited searches on Google Scholar to maximise the output of relevant publications under the given circumstances. This also explains why the number of articles detected on Google Scholar, as presented in the flow chart is larger than 1000. Also, we did not systematically search the supplementary files of articles, which may have contained information on minor parents.

Moreover, the review clarifies that information about minor parents is typically published in secondary studies and in qualitative reports on CT experiences, and not in primary CT publications. This suggested that IC information required in CT publications might be too brief to allow an adequate picture of ethical issues faced during the CT conduct and IC issues may be preferably addressed elsewhere (e.g., protocol, ethics committee review, supplementary files, or secondary article on CT challenges). Hence, future research could focus on identifying more details from screening CT protocols involving infants in SSA published in CT registries, as these may better reflect ethical considerations. However, technicalities on the identification of decision-makers may not be addressed in protocols either and may only become evident based on CT management manuals, standard operating procedures, or IC trackers, which are inaccessible to the public, if not specifically self-reported or requested.

Despite available information on the subject being rare and the related challenges to detect such information, we consider this review valuable in supporting future CT conduct. With the help of an elaborate search strategy and the unlimited consideration of various study types, we present a first overview of IC approaches applied for CT involving children with minor parents in SSA. We thereby raise evidence on the challenges faced in these situations and point to evidence-based solutions.

## Conclusions

This review highlights that there is no one-size-fits-all approach in handling IC in CTs with children of minor parents in SSA. The status of guidance is variable across countries and, frequently, clear conditions establishing minor parents’ competence to consent for themselves and their children are missing. Nevertheless, challenges can be mitigated through increasing awareness about the IC approach and appropriate planning before CT implementation. Thereby, the following should be considered: 1) Is a local law available regarding emancipation, or the “mature minor” status? 2) Does the law define whether and under what conditions minors are considered competent to consent on behalf of their children in a CT? Local laws often lack in the context of research, but when regulations on medical care exist, their provisions could also apply to research (see example by Strode and Slack (2011)), 3) Is there an existing official approach (e.g. in a national CT guideline or regulation, institutional guidance)? Did important stakeholders, including the ethics committee and the community approve the approach? Are the ministry of health, regulatory authorities, and local leaders aware of it? 4) Is the approach applicable under the individual circumstances of the CT, considering the local social and cultural context and study related risks? 5) When developing a new approach, have specific ethical considerations and practical challenges been addressed (see example provided by Ott et.al 2018 and Table 3 of this article)? 6) Was the approach described or referred to in the study protocol and were possible practical challenges mitigated? 7) Was the possibility of minor parents addressed in the CT publication? We argue that special IC situations should be described in publications and, if this is not possible due to restrictions of word count, in an appendix to the publication.

We further conclude that international CT guidelines, such as the ICH Clinical Investigation of Medicinal Products in the Pediatric Population E11 (R1), should be amended to include a general statement on the variability of IC for children of minor parents, e.g. “National guidance on the IC for children must be adhered to; where they are missing or local conventions deviate from such guidance, the process must be described in the study protocol and be mentioned in scientific publications”.

## Supporting information

S1 Checklist PRISMA checklist. (DOCX)

S1 Text Search strategies. (DOCX)

S1 Table Articles, conference abstracts, and books not analysed due to missing access or systematic exclusion. (DOCX)
