# Supplementary material for: Informed consent approaches for clinical trial participation of infants with minor parents in sub-Saharan Africa: A systematic review
Source: PLoS One. 2020 Aug 4;15(8):e0237088. doi: 10.1371/journal.pone.0237088 (PMC7402474; doi:10.1371/journal.pone.0237088)
Supplement: S1 Checklist — (DOCX) [file pone.0237088.s001.docx]

**S1 Checklist. PRISMA Checklist.**

| **Section/topic** | **#** | **Checklist item** | **Reported on page #** |
| --- | --- | --- | --- |
| **TITLE** | | |  |
| Title | 1 | Identify the report as a systematic review, meta-analysis, or both. | OK: See title (page #1). |
| **ABSTRACT** | | |  |
| Structured summary | 2 | Provide a structured summary including, as applicable: background; objectives; data sources; study eligibility criteria, participants, and interventions; study appraisal and synthesis methods; results; limitations; conclusions and implications of key findings; systematic review registration number. | OK: See (page #2-3). |
| **INTRODUCTION** | | |  |
| Rationale | 3 | Describe the rationale for the review in the context of what is already known. | OK: See Introduction starting from paragraph 2 (page #3-4). |
| Objectives | 4 | Provide an explicit statement of questions being addressed with reference to participants, interventions, comparisons, outcomes, and study design (PICOS). | OK: See last two paragraphs of the Introduction (page #4). |
| **METHODS** | | |  |
| Protocol and registration | 5 | Indicate if a review protocol exists, if and where it can be accessed (e.g., Web address), and, if available, provide registration information including registration number. | OK: See Material and methods section, first paragraph (page #4) and Search strategy section in Material and methods section, end of first paragraph (page #5) |
| Eligibility criteria | 6 | Specify study characteristics (e.g., PICOS, length of follow-up) and report characteristics (e.g., years considered, language, publication status) used as criteria for eligibility, giving rationale. | OK: See Table 1 for the search strategy (page #5-6), Table 2 about eligibility criteria in Material and methods section (page #6-7), and text (page #4-7). |
| Information sources | 7 | Describe all information sources (e.g., databases with dates of coverage, contact with study authors to identify additional studies) in the search and date last searched. | OK: See Table 1 for the search strategy (page #5-6) and Search strategy section in Material and methods section (page #4-6) |
| Search | 8 | Present full electronic search strategy for at least one database, including any limits used, such that it could be repeated. | OK: See Table 1 about search strategy in Material and methods section (page #5-6) and S1 Text. |
| Study selection | 9 | State the process for selecting studies (i.e., screening, eligibility, included in systematic review, and, if applicable, included in the meta-analysis). | OK: See Eligibility criteria and screening section in Material and methods section (page #6-8) and Table 3 about screening strategy (page #7-8). |
| Data collection process | 10 | Describe method of data extraction from reports (e.g., piloted forms, independently, in duplicate) and any processes for obtaining and confirming data from investigators. | OK: See Eligibility criteria and screening section (page #6-8), as well as Data extraction and analysis section in Material and methods section (page #8). |
| Data items | 11 | List and define all variables for which data were sought (e.g., PICOS, funding sources) and any assumptions and simplifications made. | OK: See Data extraction and analysis section in Material and methods section (page #8) and Result section (page #11). |
| Risk of bias in individual studies | 12 | Describe methods used for assessing risk of bias of individual studies (including specification of whether this was done at the study or outcome level), and how this information is to be used in any data synthesis. | OK: See Critical appraisal of studies section in Material and methods section (page #8). |
| Summary measures | 13 | State the principal summary measures (e.g., risk ratio, difference in means). | NA |
| Synthesis of results | 14 | Describe the methods of handling data and combining results of studies, if done, including measures of consistency (e.g., I^2^) for each meta-analysis. | NA |
| Risk of bias across studies | 15 | Specify any assessment of risk of bias that may affect the cumulative evidence (e.g., publication bias, selective reporting within studies). | OK: See critical appraisal of studies section in Material and methods section (page #8). |
| Additional analyses | 16 | Describe methods of additional analyses (e.g., sensitivity or subgroup analyses, meta-regression), if done, indicating which were pre-specified. | NA |
| **RESULTS** | | |  |
| Study selection | 17 | Give numbers of studies screened, assessed for eligibility, and included in the review, with reasons for exclusions at each stage, ideally with a flow diagram. | OK: See separate file for Fig 1 plus caption in Results section (page #9). |
| Study characteristics | 18 | For each study, present characteristics for which data were extracted (e.g., study size, PICOS, follow-up period) and provide the citations. | OK: See Table 4 including study characteristics in Results section (page #9-10). |
| Risk of bias within studies | 19 | Present data on risk of bias of each study and, if available, any outcome level assessment (see item 12). | NA |
| Results of individual studies | 20 | For all outcomes considered (benefits or harms), present, for each study: (a) simple summary data for each intervention group (b) effect estimates and confidence intervals, ideally with a forest plot. | OK: As far as applicable presented in table including the framework of themes concerning minor parents in Results section (page #12-17). |
| Synthesis of results | 21 | Present results of each meta-analysis done, including confidence intervals and measures of consistency. | OK: As far as applicable presented in table including the framework of themes concerning minor parents in Results section (page #12-17). |
| Risk of bias across studies | 22 | Present results of any assessment of risk of bias across studies (see Item 15). | OK: As far as applicable presented in table including the framework of themes concerning minor parents in Results section (page #12-17). |
| Additional analysis | 23 | Give results of additional analyses, if done (e.g., sensitivity or subgroup analyses, meta-regression [see Item 16]). | NA |
| **DISCUSSION** | | |  |
| Summary of evidence | 24 | Summarize the main findings including the strength of evidence for each main outcome; consider their relevance to key groups (e.g., healthcare providers, users, and policy makers). | OK: As far as applicable presented in Discussion section (page #21-25). |
| Limitations | 25 | Discuss limitations at study and outcome level (e.g., risk of bias), and at review-level (e.g., incomplete retrieval of identified research, reporting bias). | OK: See strengths and limitations section in Discussion section (page #24-25). |
| Conclusions | 26 | Provide a general interpretation of the results in the context of other evidence, and implications for future research. | OK: See Discussion and Conclusion section (page #21-25). |
| **FUNDING** | | |  |
| Funding | 27 | Describe sources of funding for the systematic review and other support (e.g., supply of data); role of funders for the systematic review. | OK: See funding information in application system. |

*From:*  Moher D, Liberati A, Tetzlaff J, Altman DG, The PRISMA Group (2009). Preferred Reporting Items for Systematic Reviews and Meta-Analyses: The PRISMA Statement. PLoS Med 6(7): e1000097. doi:10.1371/journal.pmed1000097
