# Supplementary material for: Informed consent approaches for clinical trial participation of infants with minor parents in sub-Saharan Africa: A systematic review
Source: PLoS One. 2020 Aug 4;15(8):e0237088. doi: 10.1371/journal.pone.0237088 (PMC7402474; doi:10.1371/journal.pone.0237088)
Supplement: S1 Text — (DOCX) [file pone.0237088.s002.docx]

**S1 Text. Search strategies.**

**PubMed (initial search)**

((((((((informed consent*) OR (consent form*) OR "patient information" OR (consent) OR (consented) OR (consenting) OR (assent) OR (parental permission*))) **AND** ((minor*) OR (pediatr*) OR (paediatr*) OR "child" OR "children" OR "childhood" OR infant* OR (adolescen*) OR "underage" OR (under-aged) OR (under aged*) OR (teen*) OR (minor parent*) OR (minor mother*) OR (under aged* AND parent*) OR (under-aged parent*) OR (underage parent*) OR (under aged* AND mother*) OR (under-aged mother*) OR (underage mother*) OR (teenage parent*) OR (teenage mother*) OR (adolescent parent*) OR (adolescent mother*))) **AND** ((parent*) OR "proxy" OR (representative*) OR (legally acceptable representative*) OR (guardian*) OR (caregiver*) OR (care giver*) OR (surrogate*) OR (decision making*) OR (capacity) OR (capab*) OR (competen*) OR (legal competenc*) OR (legally competent*) OR (matur*) OR (emancipat*) OR (waiv*) OR (exempt*) OR ("autonomy"))) **AND** ("trial" OR "trials" OR (clinical research*) OR "clinical trial" OR "clinical trials" OR (random*) OR "RCT" OR "pediatric research" OR "paediatric research" OR (medical research*) OR ("research" AND "child") OR ("research" AND "adolescent"))) **AND** ((low income*) OR (low resource*) OR (low-resource) OR (resource-limited) OR (resource limited*) OR (resource-poor) OR (resource poor*) OR (resource restricted*) OR (resource-restricted) OR (developing countr*) OR (global*) OR (international*) OR (developing world*) OR (less developed*) OR (less-developed) OR (less advanced*) OR (less-advanced) OR (poverty-related) OR (poverty related*) OR (LMIC*) OR "low-and middle income" OR (resource*)))) **OR** (((((("Informed Consent"[Mesh] OR "Parental Notification"[Mesh] OR "Presumed Consent"[Mesh])) **AND** ("Minors"[Mesh] OR "Child"[Mesh] OR "Infant"[Mesh] OR "Adolescent"[Mesh] OR "Child, Orphaned"[Mesh] OR "Pregnancy in Adolescence"[Mesh] OR "Maternal Age"[Mesh] OR "Vulnerable Populations"[Mesh])) **AND** ("Parents"[Mesh] OR "Legal Guardians"[Mesh] OR "Caregivers"[Mesh] OR "Decision Making"[Mesh] OR "Judicial Role"[Mesh] OR "Mental Competency"[Mesh] OR "Comprehension"[Mesh] OR "Liability, Legal"[Mesh] OR "Personal Autonomy"[Mesh] OR "Child Welfare"[Mesh] OR "Infant Welfare"[Mesh])) **AND** ("Biomedical Research"[Mesh] OR "Clinical Trials as Topic"[Mesh] OR "Research Subjects"[Mesh] OR "Pediatrics"[Mesh])) **AND** ("Developing Countries"[Mesh] OR "Poverty"[Mesh] OR "Neglected Diseases"[Mesh] OR "Culture"[Mesh] OR "Culturally Appropriate Technology"[Mesh] OR "Global Health"[Mesh] OR "Health Resources"[Mesh] OR "Global Burden of Disease"[Mesh]))

Sort by: Relevance Filters: Humans; English; French

**Embase (initial search)**

('informed consent'/syn OR 'patient information'/syn OR 'parental consent'/syn OR 'parental notification'/syn OR 'presumed consent'/syn OR 'consent' OR 'consenting' OR 'consented' OR 'assent*' OR 'parental permission' OR 'informed consent' OR 'consent form*' OR 'patient information') **AND** ('minor (person)'/syn OR 'child'/de OR 'infant'/exp OR 'adolescent'/syn OR 'parental age'/syn OR 'orphaned child'/syn OR 'preschool child'/syn OR 'school child'/syn OR 'toddler'/syn OR 'paediatrics'/de OR 'adolescent pregnancy'/syn OR 'adolescent parent'/syn OR 'vulnerable population'/syn OR 'minor*' OR 'pediatric*' OR 'paediatric*' OR 'child' OR 'children' OR 'childhood' OR 'infant*' OR 'adolescen*' OR 'adolescent patient' OR 'underage*' OR 'under age*' OR 'teen*' OR 'minor parent*' OR 'minor mother*' OR 'under aged parent*' OR 'underage parent*' OR 'under aged mother*' OR 'underage mother*' OR 'teenage parent*' OR 'teenage mother*' OR 'adolescent parent*' OR 'adolescent mother*') **AND** ('parent'/de OR 'caregiver'/syn OR 'patient decision making'/syn OR 'shared decision making'/syn OR 'ethical decision making'/syn OR 'legal liability'/syn OR 'patient autonomy'/syn OR 'competence'/syn OR 'mental capacity'/de OR 'comprehension'/syn OR 'maturity'/de OR 'child welfare'/syn OR 'custodial care'/syn OR 'child advocacy'/syn OR 'parent*' OR 'proxy' OR 'representative*' OR 'legally acceptable representative' OR 'guardian*' OR 'caregiver*' OR 'care giver*' OR 'surrogate*' OR 'decision making' OR 'capacity' OR 'capab*' OR 'competen*' OR 'legal competenc*' OR 'legally competent' OR 'matur*' OR 'emancipat*' OR 'waiv*' OR 'exempt*' OR 'autonomy') **AND** ('clinical research'/syn OR 'clinical trial'/syn OR 'clinical trial (topic)'/syn OR 'randomized controlled trial'/syn OR 'randomized controlled trial (topic)'/syn OR 'drug research'/syn OR 'research subject'/syn OR 'trial' OR 'trials' OR 'random*' OR 'rct*' OR 'clinical trial' OR 'clinical trials' OR 'clinical research' OR 'pediatric research' OR 'paediatric research' OR 'medical research' OR ('research' NEAR/3 ('child' OR 'adolescent'))) **AND** ('developing country'/syn OR 'poverty'/syn OR 'cultural factor'/syn OR 'global disease burden'/syn OR 'cultural competence'/syn OR 'neglected disease'/syn OR 'resource allocation'/syn OR 'global health'/syn OR 'low income country' OR ('resource*' NEAR/1 ('restricted' OR 'limited' OR 'low' OR 'poor')) OR 'poverty related' OR 'developing world' OR 'less developed' OR 'less advanced' OR 'lmic*' OR 'low and middle income' OR 'global*' OR 'developing countr*' OR 'international*') **AND** ([english]/lim OR [french]/lim) **AND** [humans]/lim

**Embase (updated search)**

('Informed Consent'/exp OR 'informed consent legal aspects'/exp OR 'Parental Notification'/exp OR 'patient information'/exp OR 'parental consent'/exp OR "patient information":ti,ab OR consent:ti,ab OR consented:ti,ab OR consenting:ti,ab OR assent*:ti,ab OR "parental permission*":ti,ab) **AND** ('minor (person)'/exp OR 'Child'/exp OR 'Adolescent'/exp OR 'Pediatrics'/exp OR 'adolescent pregnancy'/exp OR 'adolescent parent'/exp OR 'parental Age'/exp OR 'Vulnerable Population'/exp OR 'child health care'/exp OR 'pediatric hospital'/exp OR 'pediatric intensive care unit'/exp OR minor*:ti,ab OR pediatr*:ti,ab OR paediatr*:ti,ab OR child:ti,ab OR children:ti,ab OR childhood:ti,ab OR infant*:ti,ab OR newborn*:ti,ab OR "new born*":ti,ab OR baby:ti,ab OR babies:ti,ab OR neonat*:ti,ab OR perinat*:ti,ab OR postnat*:ti,ab OR kid:ti,ab OR kids:ti,ab OR boy*:ti,ab OR girl*:ti,ab OR preschool*:ti,ab OR kindergar*:ti,ab OR prepuberty*:ti,ab OR prepubescen*:ti,ab OR juvenil*:ti,ab OR youth*:ti,ab OR puber*:ti,ab OR pubescen*:ti,ab OR schoolchild*:ti,ab OR highschool*:ti,ab OR under-aged*:ti,ab OR underage:ti,ab OR teen*:ti,ab OR adolescen*:ti,ab) **AND** ('Parent'/exp OR 'custodial care'/exp OR 'Caregiver'/exp OR 'Decision Making'/exp OR 'jurisprudence'/exp OR 'mental capacity'/exp OR 'Comprehension'/exp OR 'legal competence'/exp OR 'legal liability'/exp OR 'patient Autonomy'/exp OR 'Child Welfare'/exp OR 'Infant Welfare'/exp OR 'maturity'/de OR 'child advocacy'/exp OR parent*:ti,ab OR proxy:ti,ab OR representative*:ti,ab OR guardian*:ti,ab OR caregiver*:ti,ab OR "care giver*":ti,ab OR surrogate*:ti,ab OR "decision making*":ti,ab OR capacity:ti,ab OR capab*:ti,ab OR competen*:ti,ab OR legal-competen*:ti,ab OR legally-competen*:ti,ab OR matur*:ti,ab OR emancipat*:ti,ab OR waiv*:ti,ab OR exempt*:ti,ab OR autonomy:ti,ab) **AND** ('medical Research'/exp OR 'Clinical Trial (topic)'/exp OR 'Research Subject'/exp OR trial:ti,ab OR trials:ti,ab OR random*:ti,ab OR RCT:ti,ab OR placebo:ti,ab OR research*:ti,ab) **AND** ('Developing Country'/exp OR 'Poverty'/exp OR 'Neglected Disease'/exp OR 'cultural factor'/exp OR 'Appropriate Technology'/exp OR 'Global Health'/exp OR 'health care planning'/exp OR 'global disease burden'/exp OR 'resource allocation'/exp OR 'cultural competence'/exp OR "low income*":ti,ab OR low-resource*:ti,ab OR resource*:ti,ab OR resource-limited:ti,ab OR resource-poor*:ti,ab OR resource-restricted:ti,ab OR "developing countr*":ti,ab OR global*:ti,ab OR international*:ti,ab OR "developing world*":ti,ab OR less-developed:ti,ab OR less-advanced:ti,ab OR poverty-related*:ti,ab OR LMIC*:ti,ab OR low-and-middle-income:ti,ab OR angola:ti,ab OR angolan:ti,ab OR benin:ti,ab OR botswana:ti,ab OR "burkina faso":ti,ab OR "upper volta":ti,ab OR burundi:ti,ab OR "côte d-ivoire":ti,ab OR "cote d-ivoire":ti,ab OR "ivory coast":ti,ab OR cameroon:ti,ab OR camerun:ti,ab OR kamerun:ti,ab OR "central african republic":ti,ab OR chad:ti,ab OR congo:ti,ab OR zaire:ti,ab OR djibouti:ti,ab OR "equatorial guinea":ti,ab OR eritrea:ti,ab OR ethiopia:ti,ab OR gabon:ti,ab OR gambia:ti,ab OR guinea:ti,ab OR "guinea bissau":ti,ab OR kenya:ti,ab OR lesotho:ti,ab OR liberia:ti,ab OR malawi:ti,ab OR mali:ti,ab OR mauritania:ti,ab OR mozambique:ti,ab OR namibia:ti,ab OR niger:ti,ab OR nigeria:ti,ab OR nigerian:ti,ab OR rwanda:ti,ab OR senegal:ti,ab OR "sierra leone":ti,ab OR somalia:ti,ab OR "south africa":ti,ab OR "south sudan":ti,ab OR sudan:ti,ab OR swaziland:ti,ab OR tanzania:ti,ab OR togo:ti,ab OR uganda:ti,ab OR zambia:ti,ab OR sambia:ti,ab OR zimbabwe:ti,ab OR rhodesia:ti,ab OR 'Africa South of the Sahara'/exp) **NOT** (('animal'/de OR 'animal experiment'/exp OR 'nonhuman'/de) **NOT** ('human'/exp OR 'human experiment'/de))

**CINAHL (initial search)**

"((MH "Consent (Research)") OR (MH "Consent") OR (MH "Protection of Human Subjects") OR (MH "Parental Notification") OR (informed consent*) OR (consent form*) OR "patient information" OR (consent) OR (consented) OR (consenting) OR (assent) OR (parental permission*) ) **AND** ( (MM "Minors (Legal)") OR (MH "Child") OR (MH "Infant") OR (MH "Child, Preschool") OR (MH "Infant, Newborn") OR (MH "Adolescence") OR (MH "Pediatrics") OR (MH "Adolescent Parents+") OR (MH "Maternal Age+") OR (MH "Adolescent Fathers") OR (MH "Adolescent Mothers") OR (MH "Special Populations") OR (minor*) OR (pediatr*) OR (paediatr*) OR "child" OR "children" OR "childhood" OR (infant*) OR (adolescen*) OR (under-aged) OR (under aged*) OR “underage” OR (teen*) OR (minor parent*) OR (under aged* AND parent*) OR (under-aged parent*) OR (underage parent*) OR (under aged* AND mother*) OR (under-aged mother*) OR (underage mother*) OR (minor mother*) OR (teenage parent*) OR (teenage mother*) OR (adolescent parent*) OR (adolescent mother*) ) **AND** ( (MH "Parents+") OR (MH "Guardianship, Legal+") OR (MH “Caregivers”) OR (MH "Decision Making, Patient") OR (MH "Decision Making, Family") OR (MH "Decision Making, Ethical") OR (MH "Liability, Legal") OR (MH "Patient Autonomy") OR (MH "Competence (Legal)") OR (MH "Child Advocacy") OR (MH "Child Custody") OR (MH "Child Welfare") OR (parent*) OR ("proxy") OR (representative*) OR (legally acceptable representative*) OR (guardian*) OR (caregiver*) OR (care giver*) OR (surrogate*) OR (decision making*) OR (capacity) OR (capab*) OR (competen*) OR (legal competenc*) OR (legally competent*) OR (matur*) OR (emancipat*) OR (waiv*) OR (exempt*) OR (“autonomy”) ) **AND** ( (MH "Experimental Studies+") OR (MH “Clinical Research”) OR (MH "Research Subjects") OR "trial" OR "trials" OR (clinical research*) OR "clinical trial" OR "clinical trials" OR (random*) OR (RCT*) OR 'pediatric research' OR 'paediatric research' OR (medical research*) OR (“research”) OR (“research” AND “child”) OR (“research AND adolescent”) ) **AND** ( (MH "Developing Countries") OR (MH "Poverty+") OR (MH "Cultural Values") OR (MH "Culture") OR (MH "Cultural Competence") OR (MH "Low and Middle Income Countries") OR (MH "Resource Allocation+") OR (MH "World Health") OR (low income*) OR (low resource*) OR (low-resource) OR (resource-limited) OR (resource limited*) OR (resource-poor) OR (resource poor*) OR (resource restricted*) OR (resource-restricted) OR (developing countr*) OR (global*) OR (international*) OR (developing world*) OR (less developed*) OR (less-developed) OR (less advanced*) OR (less-advanced) OR (poverty related*) OR (poverty-related) OR (LMIC*) OR (“low-and middle income”) OR (“resource”)) Human on 2017-07-28 05:14 AM"

**CINAHL (updated search)**

((MH "Consent (Research)") OR (MH "Consent") OR (MH "Protection of Human Subjects") OR (MH "Parental Notification") OR TI "patient information" OR AB "patient information" OR TI consent OR AB consent OR TI consented OR AB consented OR TI consenting OR AB consenting OR TI assent* OR AB assent* OR TI "parental permission*" OR AB "parental permission*") **AND** ((MH "Minors (Legal)") OR (MH "Child+") OR (MH "Adolescence+") OR (MH "Pediatrics+") OR (MH "Maternal Age+") OR (MH "Adolescent Parents+") OR (MH "Special Populations") OR (MH "Child Health Services+") OR (MH "Hospitals, Pediatric") OR (MH "Intensive Care Units, Pediatric+") OR TI minor* OR AB minor* OR TI pediatr* OR AB pediatr* OR TI paediatr* OR AB paediatr* OR TI child OR AB child OR TI children OR AB children OR TI childhood OR AB childhood OR TI infant* OR AB infant* OR TI newborn* OR AB newborn* OR TI "new born*" OR AB "new born*" OR TI baby OR AB baby OR TI babies OR AB babies OR TI neonat* OR AB neonat* OR TI perinat* OR AB perinat* OR TI postnat* OR AB postnat* OR TI kid OR AB kid OR TI kids OR AB kids OR TI boy* OR AB boy* OR TI girl* OR AB girl* OR TI preschool* OR AB preschool* OR TI kindergar* OR AB kindergar* OR TI prepuberty* OR AB prepuberty* OR TI prepubescen* OR AB prepubescen* OR TI juvenil* OR AB juvenil* OR TI youth* OR AB youth* OR TI puber* OR AB puber* OR TI pubescen* OR AB pubescen* OR TI schoolchild* OR AB schoolchild* OR TI highschool* OR AB highschool* OR TI under-aged* OR AB under-aged* OR TI underage OR AB underage OR TI teen* OR AB teen* OR TI adolescen* OR AB adolescen*) **AND** ((MH "Parents+") OR (MH "Guardianship, Legal+") OR (MH "Caregivers") OR (MH "Decision Making+") OR (MH "Jurisprudence+") OR (MH "Autonomy+") OR (MH "Child Welfare+") OR (MH "Child Advocacy") OR TI parent* OR AB parent* OR TI proxy OR AB proxy OR TI representative* OR AB representative* OR TI guardian* OR AB guardian* OR TI caregiver* OR AB caregiver* OR TI "care giver*" OR AB "care giver*" OR TI surrogate* OR AB surrogate* OR TI "decision making*" OR AB "decision making*" OR TI capacity OR AB capacity OR TI capab* OR AB capab* OR TI competen* OR AB competen* OR TI legal-competen* OR AB legal-competen* OR TI legally-competen* OR AB legally-competen* OR TI matur* OR AB matur* OR TI emancipat* OR AB emancipat* OR TI waiv* OR AB waiv* OR TI exempt* OR AB exempt* OR TI autonomy OR AB autonomy) **AND** ((MH "Research, Medical") OR (MH “Clinical Research”) OR (MH "Research Subjects+") OR TI trial OR AB trial OR TI trials OR AB trials OR TI random* OR AB random* OR TI RCT OR AB RCT OR TI placebo OR AB placebo OR TI research* OR AB research*) **AND** ((MH "Developing Countries") OR (MH "Poverty") OR (MH "Neglected Diseases") OR (MH "Culture+") OR (MH "Cultural Competence") OR (MH "Low and Middle Income Countries") OR (MH "Resource Allocation+") OR (MH "World Health") OR TI "low income*" OR AB "low income*" OR TI low-resource* OR AB low-resource* OR TI resource* OR AB resource* OR TI resource-limited OR AB resource-limited OR TI resource-poor* OR AB resource-poor* OR TI resource-restricted OR AB resource-restricted OR TI "developing countr*" OR AB "developing countr*" OR TI global* OR AB global* OR TI international* OR AB international* OR TI "developing world*" OR AB "developing world*" OR TI less-developed OR AB less-developed OR TI less-advanced OR AB less-advanced OR TI poverty-related* OR AB poverty-related* OR TI LMIC* OR AB LMIC* OR TI low-and-middle-income OR AB low-and-middle-income OR TI angola OR AB angola OR TI angolan OR AB angolan OR TI benin OR AB benin OR TI botswana OR AB botswana OR TI "burkina faso" OR AB "burkina faso" OR TI "upper volta" OR AB "upper volta" OR TI burundi OR AB burundi OR TI "côte d’ivoire" OR AB "côte d’ivoire" OR TI "cote d’ivoire" OR AB "cote d’ivoire" OR TI "ivory coast" OR AB "ivory coast" OR TI cameroon OR AB cameroon OR TI camerun OR AB camerun OR TI kamerun OR AB kamerun OR TI "central african republic" OR AB "central african republic" OR TI chad OR AB chad OR TI congo OR AB congo OR TI zaire OR AB zaire OR TI djibouti OR AB djibouti OR TI "equatorial guinea" OR AB "equatorial guinea" OR TI eritrea OR AB eritrea OR TI ethiopia OR AB ethiopia OR TI gabon OR AB gabon OR TI gambia OR AB gambia OR TI guinea OR AB guinea OR TI "guinea bissau" OR AB "guinea bissau" OR TI kenya OR AB kenya OR TI lesotho OR AB lesotho OR TI liberia OR AB liberia OR TI malawi OR AB malawi OR TI mali OR AB mali OR TI mauritania OR AB mauritania OR TI mozambique OR AB mozambique OR TI namibia OR AB namibia OR TI niger OR AB niger OR TI nigeria OR AB nigeria OR TI nigerian OR AB nigerian OR TI rwanda OR AB rwanda OR TI senegal OR AB senegal OR TI "sierra leone" OR AB "sierra leone" OR TI somalia OR AB somalia OR TI "south africa" OR AB "south africa" OR TI "south sudan" OR AB "south sudan" OR TI sudan OR AB sudan OR TI swaziland OR AB swaziland OR TI tanzania OR AB tanzania OR TI togo OR AB togo OR TI uganda OR AB uganda OR TI zambia OR AB zambia OR TI sambia OR AB sambia OR TI zimbabwe OR AB zimbabwe OR TI rhodesia OR AB rhodesia OR (MH "Africa South of the Sahara+")) **NOT** ((MH "animals+") **NOT** (MH "humans+"))

**Google Scholar (initial search)**

1. consent ("minor parent" OR parental OR "adolescent parent" OR "teenage parent") (representative OR proxy OR guardian OR “caregiver” OR surrogate OR mature OR emancipated OR autonomy) (“clinical trial" OR "clinical trials") "developing countries"

2. consent (“adolescent mother” OR "minor mother" OR "teenage mother" OR "underage mother") (“clinical trial" OR "clinical trials") "developing countries"

3. Search for related papers of “McAdams JJ 2013, Determining the Consenting Capacity of Minors”

Limit all searches to languages (Engl & French)

**Google Scholar (updated search)**

1. consent ("minor parent" OR parental OR "adolescent parent" OR "teenage parent") (representative OR proxy OR guardian OR “caregiver” OR surrogate OR mature OR emancipated OR autonomy) (“clinical trial" OR "clinical trials") "developing countries"

2. consent (“adolescent mother” OR "minor mother" OR "teenage mother" OR "underage mother") (“clinical trial" OR "clinical trials") "developing countries"

3. Search for related papers of “McAdams JJ 2013, Determining the Consenting Capacity of Minors”

No limitations
