# Supplementary material for: Informed consent approaches for clinical trial participation of infants with minor parents in sub-Saharan Africa: A systematic review
Source: PLoS One. 2020 Aug 4;15(8):e0237088. doi: 10.1371/journal.pone.0237088 (PMC7402474; doi:10.1371/journal.pone.0237088)
Supplement: S1 Table — (DOCX) [file pone.0237088.s003.docx]

### S5 Table. Articles, conference abstracts, and books not analysed due to missing access or systematic exclusion.

| **Author** | **Titel** | **Journal/Publisher** | **Access** | **Type** |
| --- | --- | --- | --- | --- |
| (1974) | Biomedical technology - the ethical dilemmas | JSAC Grapevine | No access | Journal Article |
| (1993) | Recommendations of the International Symposium on Contraceptive Research and Development for the Year 2000 and Beyond | Progress in Human Reproduction Research | No access | Conference Abstract |
| Abdulah Aziz et al. (2011) | Acceptability of short-course AZT prevention regimen by HIV infected pregnant women; should VCT in the Antenatal setting be modified | Sexually Transmitted Infections | No access | Conference Abstract |
| Addissie and Mitiku (2017) | Validating and promoting 'rapid ethical assessment' as a practical method for enhancing ethical conduct of tropical disease research projects in developing countries | Tropical Medicine and International Health | No access | Conference Abstract (Poster) |
| Anne-Laure et al. (2009) | Clinical research in less economically developed countries: The ethical challenges | Tropical Medicine and International Health | No access | Conference Abstract |
| Bachenheimer and Brescia (2007) | Reinventing patient recruitment: revolutionary ideas for clinical trial success | Gower Publishing, Ltd. | No access | Book |
| Ballantyne and Rogers (2016) | Pregnancy, vulnerability, and the risk of exploitation in clinical research | Springer |  | Book Section |
| Barnabas et al. (2008) | HIV/AIDS Vaccine Research | Springer | No access | Book Section |
| Barrett et al. (2016) | Public health research | Springer | No access | Book Section |
| Baud et al. (2017) | Dosage regimen of biperiden to treat haloperidol-induced severe facio-troncular dystonic syndrome in children | Annals of Intensive Care | No access | Conference Abstract |
| Behrman and Field (2004) | Ethical conduct of clinical research involving children | National Academies Press |  | Book (Booklet) |
| Benedetti and Kesselheim (2016) | Ethics in Pediatric Oncology | Springer |  | Book Section |
| Benson and Roth (1988) | Trends in the social control of medical and psychiatric research | Law and mental health |  | Book Section |
| Bentley et al. (2013) | The breastfeeding, antiretrovirals, and nutrition (BAN) study in Malawi: Use of qualitative methods to guide study design and evaluation of a randomized controlled trial | Annals of Nutrition and Metabolism | No access | Conference Abstract |
| Berg (2005) | Children and placebos | Ethics and Research with Children: A Case-Based Approach |  | Book |
| Berman and Field (2004) | The ethical conduct of clinical research involving children | National Academies Press |  | Book |
| Bhutta and Offringa (2015) | Standards of Research for Clinical Trials in Low-and Middle-Income Countries | Springer |  | Book |
| Bogie et al. (2015) | Non-invasive haemoglobin measurements for assessing anaemia in Kenyan school children as part of an integrated school health and nutrition programme | Archives of Disease in | No access | Conference Abstract |
| Bonsall et al. (2018) | HIV genotyping and phylogenetics in the HPTN 071 (PopART) study: Validation of a high-throughput sequencing assay for viral load quantification, genotyping, resistance testing and high-resolution transmission networking | Journal of the International AIDS Society | No access | Conference Abstract |
| Caporale and Pavone (2018) | International Biolaw and Shared Ethical Principles (by Caporale), The Universal Declaration on Bioethics and Human Rights as a landmark in the development of global bioethics (by Henk Ten Have) | Routledge | No access | Book Section |
| Chaponda (2012) | Ethical challenges in the conduct of the study-evaluation of four artemisinin-based combinations for the treatment of uncomplicated malaria in African children at the Ndola site | Tropical Medicine and Health | No access | Conference Abstract |
| Chi et al. (2014) | Risk-Benefit Assessment | Research Ethics in Africa: A Resource for Research Ethics committees |  | Book |
| Chi et al (2014) | 10 Risk‑benefit Assessment | - |  | Book |
| Clarke et al. (2005) | Research with children | A Handbook of Research Methods for Clinical and Health Psychology | No access | Book Section |
| Clarke et al. (2012) | A new approach for malaria control in schools: Results of a randomized trial of intermittent parasite clearance | American Journal of Tropical Medicine and Hygiene | No access | Conference Abstract |
| Close (2010) | The effect of probiotics in reducing the duration of acute infectious diarrhea in children: a literature review | International Journal of Probiotics & Prebiotics | No access | Journal Article |
| Coffin and Nelson (2005) | Optimizing Risks and Benefits: The Case of Rotavirus Vaccine | Ethics and Research with Children: A Case-Based Approach | No access | Book Section |
| Corneli and Borasky (2014) | Research Ethics and Working With Institutional Review Boards | Public Health Research Methods | No access | Book Section |
| Coughlin (2013) | Ethical issues in cancer epidemiologic studies | Cancer Epidemiology: Low-and Middle-Income Countries and Special Populations |  | Book |
| Darabi et al. (2013) | The effect of vitamin D supplementation over asthma outcome | Iranian Journal of Allergy, Asthma and Immunology | No access | Journal Article |
| de Zulueta (-) | HIV in pregnancy: ethical issues in screening and therapeutic research | Maternal-fetal medicine | No access | Journal Article |
| Dhai (2002) | Clinical Research in Africa | - | No access | Journal Article |
| Dickert and Grady (2008) | Incentives for research participants | Clinical Research Ethics | No access | Book Section |
| Duke (2016) | Randomised trials in child and adolescent health in developing countries | - |  | Book |
| Ferris and Marquis (2005) | Bioethics in scientific research: Conflicts between subject's equitable access to participate in research and current regulations | The Journal of nutrition | No access | Symposium Abstract |
| Field and Behrman (2004) | The necessity and challenges of clinical research involving children | Institute of Medicine Committee on Clinical Research Involving Children |  | Book |
| Field and Boat (2012) | Ethical Issues in Pediatric Drug Studies | - |  | Book |
| Fletcher (1995) | Gene therapy in mental retardation: ethical considerations | Mental retardation and developmental disabilities research reviews | No access | Journal Article |
| Fletcher and Richter (1996) | Ethical issues of perinatal human gene therapy | J Matern Fetal Med | No access | Journal Article |
| Foxcroft (2017) | Ethical Issues in Conducting Child Development Research in Sub-Saharan Africa | Springer | No access | Book Section |
| Friele (2012) | 3.2 Die Deklaration von Helsinki und die Regelung des Informed consent–Zur Berücksichtigung interkultureller Aspekte nach der Revision von | Die Deklaration von Helsinki: Revisionen und Kontroversen | No access | Book Section |
| Gangestad and Salata (2012) | Ethical issues in microbicide clinical trials for HIV prevention | Current HIV research | No access | Journal Article |
| Glover and Nwomeh (2017) | Ethical Considerations in Pediatric Surgery | Pediatric Surgery |  | Book Section |
| Goodman and Prineas (1996) | Toward an ethics curriculum in epidemiology | Ethics and epidemiology | No access | Book |
| Halac et al. (2017) | Workshop: “clinical research in vulvar disease: A multidisciplinary approach” | Journal of Lower Genital Tract Disease | No access | Workshop Abstract |
| Hamed (2007) | Early Delivery versus Expectant Management in Patients with Preterm Prelabour Rupture of Membranes at 34-37 weeks of Gestation. | The scientific J of Elminia Faculty of medicine | No access | Journal Article - Arabic |
| Hartnett (2011) | Minority research: building trust project aims to improve participation and strengthen capacity for investigators and IRBs | Research Practitioner | No access | Journal Article |
| Helmchen et al. (2014) | From exclusion to inclusion: improving clinical research in vulnerable populations; memorandum | - |  | Book |
| Hicks (2015) | Ethical and regulatory considerations in the design of traumatic brain injury clinical studies | Elsevier | No access | Book Section (Handbook) |
| Holzemer (2010) | Responsible conduct of research | Improving health through nursing research |  | Book Section |
| Hurst and Elger (2011) | New issues facing IRBs | J Med |  | Book Section |
| Ibia and Binkowitz (2016) | General Principles and Considerations in Multiregional Clinical Trials for Simultaneous Global New Drug Development | Multiregional Clinical Trials for Simultaneous Global New Drug Development | No access | Book Section |
| Ip (2016) | A Relational Account of Global Egalitarian Justice | Springer | No access | Book |
| Kahn et al. (1998) | Beyond consent: Seeking justice in research | Oxford University Press on Demand | No access | Book |
| Kartikeyan et al. (2007) | Human Rights, Legal, and Ethical Issues | HIV and AIDS: Basic Elements and Priorities | No access | Book Section |
| Kasule et al. (1995) | A clinical trial of Exluton, a progestogen only contraceptive pill containing 0.5mg lynestrenol amongst lactating Zimbabwean women | British Journal of Family Planning | No access | Journal Article |
| King and Nicholson (1986) | Informed consent | Bulletin (Institute of Medical Ethics (Great Britain)) | No access | Journal Article |
| Kleiderman and Knoppers (2017) | Minors and incompetent adults: A tale of two populations | Neuroethics: Anticipating the Future | No access | Book Section |
| Knoppers and Sprumont (2000) | Human Subjects Research, Ethics, and International Codes on Genetic Research | Encyclopedia of Ethical, Legal and Policy Issues in Biotechnology |  | Book |
| Koch and Raschka (2002) | Ethical principles for clinical trials in children | MMW-Fortschritte der Medizin | No access | Journal Article |
| Kodish (2018) | Oncology Group from 2002 to 2008 and was an appointed member of the Committee on Bioethics of the American Academy of Pediatrics from 1999 | Ethics and Research with Children: A Case-Based Approach (2^nd^ Edition) | No access | Book |
| Kopelman (2000) | Human Subjects Research, Ethics, and Research on Children | Encyclopedia of Ethical, Legal and Policy Issues in Biotechnology |  | Book |
| Kruger (2010) | Ethical issues in clinical trials in the developing world | Pediatric Blood and Cancer | No access | Conference Abstract |
| Kruse-Jarres et al. (2013) | Regional factors influencing participation in clinical trials in hemophilia in the United States of America and South Africa | Journal of Thrombosis and Haemostasis | No access | Conference Abstract |
| Lambert and Barry (2003) | 10 Future challenges for vaccines | The Vaccine Book | No access | Book |
| Levison and Levison (2001) | Women's health and human rights | Women, Gender, and Human Rights: A Global Perspective, ed. Marjorie Agosin | No access | Book |
| Lo et al. (2001) | Addressing ethical issues | Designing clinical research |  | Book |
| Loue and Okello (2000) | Research bioethics in the Ugandan context. II: Procedural and substantive reform | The Journal of law, medicine & ethics: a journal of the American Society of Law, Medicine & Ethics |  | Book Section |
| Loue (2002) | Governing Principles | Legal and Ethical Aspects of HIV-Related Research |  | Book Section |
| MacDonald et al. (2019) | 193. Ethical Aspects of Involving Children And Adolescents In HIV Research: A Systematic Review of The Empiric Literature | Journal of Adolescent Health | No access | Conference Abstract |
| Maklehemena (1998) | Voices of women | Integration | No access | Conference Abstract |
| Manning et al. (2009) | Ethical implications of informed consent in emergent clinical situations in a “Bush Hospital” in Mali | American Journal of Tropical Medicine and Hygiene | No access | Conference Abstract |
| Maple et al. (2018) | Assessing strategies and capacity for gender integration in HIV biomedical research | AIDS Research and Human Retroviruses | No access | Conference Abstract (Poster) |
| Marshall (2007) | Ethical challenges in study design and informed consent for health research in resource-poor settings | World Health Organization |  | Book |
| Mathuna (2012) | Ethical considerations in designing intervention studies | Intervention research: Designing, conducting, analyzing and funding |  | Book Section |
| Maticka-Tyndale (2004) | Dilemmas for obtaining consent when working with children in high AIDS prevalence regions | NCEHR Commun | No access | Journal Article |
| Miller (2007) | Ethical considerations in multiple sclerosis clinical trials | Multiple Sclerosis Therapeutics, 3rd ed. London, UK: Informa Healthcare |  | Book Section |
| Miller (2005) | Ethics in Research | Wiley StatsRef: Statistics Reference Online | No access | Book |
| Moin (2013) | New ethical and legal challenges and issues in pediatrics | Iranian Journal of Allergy, Asthma and Immunology | No access | Conference Abstract |
| Mulberg et al. (2013) | Pediatric drug development | John Wiley & Sons | No access | Book |
| Munir and Earls (1992) | Ethical principles governing research in child and adolescent psychiatry | Journal of the American Academy of Child & Adolescent Psychiatry | No access | Journal Article |
| Munro (2018) | 18.6 Conducting Interventions in Acute Care Settings | Intervention Research and Evidence-Based Quality Improvement: Designing, Conducting, Analyzing, and Funding | No access | Book |
| Mwale (2017) | Risk, Rewards, and Rational Consent in Healthy Volunteering | Springer |  | Book Section |
| Nakalega et al. (2018) | Ethical considerations for involving adolescents aged 16-17 in HIV prevention clinical trials: Community perspectives from Uganda | AIDS Research and Human Retroviruses | No access | Conference Abstract |
| Nalugoda et al. (2009) | Is there coercion or undue inducement to participate in health research in developing countries? An example from Rakai, Uganda | Journal of Clinical Ethics | No access | Journal Article |
| Nelson and Classic (2015) | Informed Consent in Radiation Medicine Practice and Research | Radiation Protection in Medical Imaging and Radiation Oncology |  | Book Section |
| Nelson and Roth-Cline (2015) | Ethical considerations in the design of pediatric clinical trials in low-and middle-income countries | Springer |  | Book |
| O’Mathúna (2018) | Ethical considerations in designing intervention studies | Intervention Research and Evidence-Based Quality Improvement: Designing, Conducting, Analyzing, and Funding | No access | Book Section |
| Obi (2017) | Global Clinical Research | Springer | No access | Book Section |
| Oluremi and Moses (2016) | Knowledge and willingness of young adults to participate in early HIV vaccine trials and contraceptive practices in Southwestern, Nigeria | AIDS Research and Human Retroviruses | No access | Conference Abstract (Poster) |
| Onwuatuelo (2012) | Adolescents' perception of HIV vaccine trials in Nigeria | Tropical Medicine and Health | No access | Conference Abstract |
| Ott et al. (2013) | Ethics and Vulnerability in International Research with Adolescents | Turkish Archives of | No access | Journal Article |
| Pedreira Massa (1998) | Research activities in child and adolescent psychiatry: Ethical approach and informed consent | Anales de Psiquiatria | No access | Journal Article |
| Perrey and Ymba (2009) | From information to the decision: the motives of consent to a vaccine trial for hepatitis B in the Ivory Coast | Journal de bioéthique |  | Book Section |
| Petrini (2016) | 8 Ethical and deontological issues in paediatric clinical studies: An analysis of documents from national and international institutions | Neurotechnology and Direct Brain Communication: New insights and responsibilities concerning speechless but communicative subjects | No access | Book |
| Plomer (2013) | The law and ethics of medical research: international bioethics and human rights | Routledge-Cavendish | No access | Book |
| Pope (2010) | Legal briefing: Informed consent | Journal of Clinical Ethics | No access | Journal Article |
| Powers et al. (2010) | Patient and parent awareness and concerns with participation in clinical trials | Pediatric Pulmonology | No access | Conference Abstract |
| Rahimzadeh et al. (2017) | Minors and incompetent adults: A tale of two populations | Neuroethics: Anticipating the future | No access | Book Section |
| Ravinetto et al. (2016) | Pooling knowledge and experience to improve clinical research standards in low-and middle-income countries: The experience of the switching the poles network (2008-2016) | American Journal of Tropical Medicine and Hygiene | No access | Conference Abstract |
| Ravinetto et al. (2009) | Informed consent, decision-making capacity and vulnerability in resource-constrained settings | Tropical Medicine and International Health | No access | Conference Abstract |
| Ravitsky et al. (2009) | The Penn Center Guide to Bioethics | Springer Publishing Company | No access | Book |
| Rivera et al. (2009) | Research ethics training curriculum | Academy for Educational Dev |  | Book |
| Rogowska-Szadkowska and Chlabicz (2008) | Microbicides in HIV infection prophylaxis–not only ethical challenges | HIV & AIDS Review | No access | Journal Article |
| Roth-Cline et al. (2011) | Ethical considerations in conducting pediatric research | Springer |  | Book |
| Royer (1982) | Ethics and pediatrics | Concours Medical | No access | Journal Article |
| Hurst and Elger (2010) | Research and publication | Clinical Ethics in Anesthesiology: A Case-Based Textbook | No access | Book Section |
| Sass (2006) | Towards Risk Factor Health Assessment and Education | Springer |  | Book |
| Sass (1988) | Dependency on raw nature by building homes, farms, and machines, by healing diseases and developing drugs to treat them, and by establishing | The Use of Human Beings in Research: With Special Reference to Clinical Trials | No access | Book |
| Simar and Fowler (2010) | Consent and Assent in Paediatric Clinical Trials | Karger Publishers |  | Book Section |
| Simon (-) | Clinical Research Ethics | Clinical Research | No access | Journal Article |
| Sirisena et al. (2016) | The Provision of Medical and Health Genetics and Genomics in the Developing World | Elsevier | No access | Book Section |
| Slack et al. (2003) | Guidelines on Ethics for Medical Research | South African Medical Research Council |  | Book |
| Sullivan et al. (2017) | Malawian women's experiences of rules regarding participation in HIV prevention and treatment clinical trials during pregnancy | American Journal of Obstetrics and Gynecology | No access | Conference Abstract (Poster) |
| Thorne (1997) | Vancouver Summaries: Children | AIDS Care | No access | Journal Article |
| Thornthwaite et al. (2016) | Peroxybioflavonoids (MALSUP): A possible cure for severe cases of plasmodium falciparum malaria infection in Nigeria | European Journal of Immunology | No access | Conference Abstract |
| Totri and Eichenfield (2015) | Clinical Research in Pediatric Dermatology | Springer |  | Book |
| Tremellen and Belford (2010) | Ethical Issues in Clinical Research | Pharmaceutical Sciences Encyclopedia | No access | Book |
| Urato and Lurie (-) | Health and Safety | - | No access | Journal Article |
| Van Den Bent et al. (2017) | Evaluation of depatuxizumab mafodotin (ABT-414) in children with high grade glioma (HGG) and diffuse intrinsic pontine glioma (DIPG) | Neuro-Oncology | No access | Conference Abstract |
| VanGeest and Cummins (2008) | International Research Ethics | Global Health Care: Issues and Policies | No access | Book |
| Wasunna and Bukusi (2014) | 6 A Stepwise Approach to Protocol Review | Research Ethics in Africa: A Resource for Research Ethics committees |  | Book |
| Watcha et al. (2010) | Validation of the baxter animated retching faces (BARF) scale for measuring nausea in children | Anesthesia and Analgesia | No access | Journal Article |
| Wawer (2001) | Developing Countries | Epidemiologic Methods for the Study of Infectious Diseases |  | Book |
| Weeks (2012) | Community-based use of misoprostol for pph prevention: Snapshot from a pilot study in Uganda documenting self-administration of misoprostol in a home delivery setting | International Journal of Gynecology and Obstetrics | No access | Conference Abstract (Poster) |
| Williams (2010) | Ethical Challenges in HIV Research and Clinical Care | HIV/AIDS Related Communication, Hearing and Swallowing Disorders | No access | Book Section |
| Williams (2014) | Capacity building in cancer management in Africa: Envisioning a future from past challenges | Cancer Research | No access | Conference Abstract |
| Wright and de Chesnay (2015) | Research with Vulnerable Populations: Implications for Developed and Developing Countries | Caring for the Vulnerable | No access | Book Section |
